# Copper–zinc superoxide dismutase (Sod1) activation terminates interaction between its copper chaperone (Ccs) and the cytosolic metal-binding domain of the copper importer Ctr1

**DOI:** 10.1007/s10534-019-00206-3

**Published:** 2019-07-10

**Authors:** Amélie Skopp, Stefanie D. Boyd, Morgan S. Ullrich, Li Liu, Duane D. Winkler

**Affiliations:** Department of Biological Sciences, The University of Texas at Dallas, 800 W. Campbell Road, Richardson, TX 75080 USA

**Keywords:** Ctr1, Sod1, Ccs, Copper-trafficking, Copper chaperone

## Abstract

Copper–zinc superoxide dismutase (Sod1) is a critical antioxidant enzyme that rids the cell of reactive oxygen through the redox cycling of a catalytic copper ion provided by its copper chaperone (Ccs). Ccs must first acquire this copper ion, directly or indirectly, from the influx copper transporter, Ctr1. The three proteins of this transport pathway ensure careful trafficking of copper ions from cell entry to target delivery, but the intricacies remain undefined. Biochemical examination of each step in the pathway determined that the activation of the target (Sod1) regulates the Ccs·Ctr1 interaction. Ccs stably interacts with the cytosolic C-terminal tail of Ctr1 (Ctr1c) in a copper-dependent manner. This interaction becomes tripartite upon the addition of an engineered immature form of Sod1 creating a stable Cu(I)-Ctr1c·Ccs·Sod1 heterotrimer in solution. This heterotrimer can also be made by the addition of a preformed Sod1·Ccs heterodimer to Cu(I)-Ctr1c, suggestive of multiple routes to the same destination. Only complete Sod1 activation (i.e. active site copper delivery and intra-subunit disulfide bond formation) breaks the Sod1·Ccs·Ctr1c complex. The results provide a new and extended view of the Sod1 activation pathway(s) originating at cellular copper import.

**Electronic supplementary material:**

The online version of this article (10.1007/s10534-019-00206-3) contains supplementary material, which is available to authorized users.

## Introduction

Copper is a critical cofactor for many enzymes that take advantage of its redox activity to catalyze a wide range of chemical reactions. Proper levels of copper are required throughout the cell for processes such as respiration, enzymatic catalysis, and neurotransmitter synthesis (Bremner 1998). Unregulated copper-based reactions are detrimental to the cell by generating free radical oxygen species (Halliwell and Gutteridge 1990; Pena et al. 1999). Therefore, aerobic organisms have evolved a tightly controlled network of copper trafficking molecules for import, shuttling, and delivery to specific targets across the cellular landscape (reviewed in Rosenzweig and O’Halloran 2000; Rosenzweig 2001; Robinson and Winge 2010).

Copper ion import is facilitated by the Ctr family of integral membrane transporters that bring copper into the cytoplasm in an ATP-independent fashion (Dancis and Haile 1994; Lee et al. 2002; Kim et al. 2008). Members of the Ctr-family, of which Ctr1 is the most prominent, are small transmembrane proteins consisting of a conserved extracellular N-terminal domain (ecto/NTD), three transmembrane domains (TMDs), and a short C-terminal tail (Ctr1c), which is the only domain that fully extends into the cytosol (Eisses and Kaplan 2002; Puig et al. 2002; Klomp et al. 2003). Trimerization of Ctr1 monomers forms a pore through which Cu(I) (i.e. reduced/cuprous copper) is transported into the cell (De Feo et al. 2009). Cu(I) is then simultaneously coordinated by three Ctr1c tails, each containing an HCH copper binding motif. Binding of Cu(I) to a single HCH at one tail is possible, however normal Cu(I)-binding motifs involve two or three cysteine residues coordinating Cu(I). (Pickering et al. 1993) By holding the Cu(I) between multiple tails, Ctr1 can mimic the more favorable Cu(I) coordinating motifs. The Cu(I) is then offered to cytosolic copper traffickers (Kahra et al. 2016). Two ATP-dependent pumps ATP7A and ATP7B that reside in the membranes of the trans-Golgi network, handle copper ion export (Lutsenko et al. 2002; Voskoboinik and Camakaris 2002).

A diverse family of intracellular copper binding proteins termed “copper chaperones” transport cytosolic copper ions to dedicated protein targets (O’Halloran and Culotta 2000; Kahra et al. 2016; Kaplan and Maryon 2016). One such molecule is the copper chaperone for Sod1 (Ccs) that has been shown to be critical for efficient activation of the ubiquitous anti-oxidant enzyme copper–zinc superoxide dismutase (Sod1) (Culotta et al. 1997; Schmidt et al. 2000; Wong et al. 2000; Fetherolf et al. 2017a, b). Ccs proteins consist of three conserved domains that are essential for target recognition and copper ion delivery (reviewed in Fetherolf et al. 2017a, b). Unlike other copper chaperones, Ccs also plays a crucial role in the formation of an intra-subunit disulfide bond within Sod1 (reviewed in Fetherolf et al. 2017a, b). The N-terminal domain (D1) contains a MxCxxC copper-binding motif and is structurally similar to another copper chaperone that guides copper to the aforementioned secretory pathway (Atx1/Atox1) (Xiao and Wedd 2002; Hussain et al. 2008; Rodriguez-Granillo and Wittung-Stafshede 2008; Hussain et al. 2009; Rodriguez-Granillo and Wittung-Stafshede 2009; Rodriguez-Granillo et al. 2010). The second domain (D2) is analogous to Sod1 in both sequence and structure (Lamb et al. 1999) and plays a key role in Sod1 binding (Lamb et al. 2000, 2001; Winkler et al. 2009; Fetherolf et al. 2017a, b). The C-terminal domain (D3) possesses an invariant CxC motif known to bind copper and is essential for complete Sod1 activation in vivo (Schmidt et al. 1999). Though multiple three-dimensional structures of Ccs (Lamb et al. 1999, 2000; Banci et al. 2013) and complete Ccs·Sod1 complexes have been determined (Lamb et al. 2001; Fetherolf et al. 2017a, b; Sala et al. 2019) a complete molecular mechanism for Ccs-mediated Sod1 activation is tenuous and remains under debate.

Even less understood is how, where, or when copper chaperones, including Ccs, acquire their copper cargo from the cell (Flores and Unger 2013). Indeed, the passage of Cu(I) from the cytosolic Ctr1c domain to Ccs still requires clarity, while related work implies that the reduced form of glutathione (GSH) may play an intermediary role (Maryon et al. 2013; Pope et al. 2013). Although Ctr1 has been studied extensively for years, an initial model for Ctr1-mediated Cu(I) import is only beginning to emerge (reviewed in Ohrvik and Thiele 2014).

Here, we demonstrate a direct Cu(I)-dependent interaction between human Ctr1c and Ccs. Unexpectedly, immediate copper transfer and dissolution of the Cu(I)-Ctr1c·Ccs complex did not occur. Furthermore, a stable Cu(I)-Ctr1c·Ccs·Sod1 heterotrimeric complex is observed when copper delivery and/or disulfide bond formation in Sod1 (i.e. Sod1 activation) is stalled. Cu(I)-Ctr1c will readily bind either Ccs or a pre-formed Ccs·Sod1 complex, which backs related data showing that the copper binding status of Ccs does not affect its affinity for immature Sod1 (Luchinat 2017; Boyd et al. 2018). Complete Sod1 activation (i.e. zinc and copper binding along with disulfide oxidation (Cu,Zn-Sod1^SS^)) dissociates the heterotrimeric complex into a mixture of its own parts (Banci et al. 2012; Wright et al. 2016). The data presented here builds a comprehensive molecular mechanism for the direct Ctr1-to-Ccs-to-Sod1 copper influx pathway and suggests that the entire pathway can proceed within a single macromolecular complex.

## Materials and methods

### Materials

The C-terminal 13 amino acids of the human Ctr1 Cu(I)-transporter (Ctr1c), KKAVVVDITEHCH, were custom-ordered as a lyophilized peptide from Sigma. DTT (dithiotreitol), TCEP-HCl (tris(2-carboxyethyl)phosphine), and IPTG (isopropyl 1-thio-β-d-galactopyranoside) were purchased from GoldBio. Tris-base, mono- and dibasic sodium phosphate, sodium acetate, ammonium persulfate (APS), EDTA, β-mercaptoethanol (BME), and sodium chloride were acquired from Fisher. Nitroblue terazolium and bathocuproinedisulfonic acid (BCS) were purchased from Alfa Aesar. Primers for site-directed mutagenesis and zinc sulfate heptahydrate were bought from Sigma. Maleimide-polyethyleneglycol 2000 was purchased from nanocs. Tetramethylethylenediamine (TEMED) was purchased from Thermo Scientific, while riboflavin was acquired from Acros Organics. Alexa-488-succinimidyl ester was purchased from Life Technologies. Cu(I)-(CH_3_CN_4_)PF_6_ was purchased from Strem Chemicals. His-Trap Nickel affinity columns, SQ (anion exchange) columns, and gel filtration columns were purchased from GE LifeSciences.

### Ccs1 and Sod1 cloning, mutagenesis, expression, and purification

Human wild-type (WT) Ccs1 (UniProtKB—O14618) was cloned into a pAG8H vector containing an inducible *lacZ* site, N-terminal His_8_-tag, and an internal tobacco etch virus (TEV) cleavage site using *NarI* and *SalI* restriction sites. Sod1 WT (UniProtKB—P00441) was cloned using *NarI* and *HindIII* sites into the same vector. Mutations in Ccs and Sod1 were generated using the Thermo Scientific site-directed mutagenesis kit according to the provided protocol. *Escherichia coli* BL21 DE3 PLysS cells (purchased from Promega) were transformed and grown at 37 °C in 2xYT medium to an A_600nm_ of 0.6–0.9 and induced with 3–5 mM IPTG. After an additional 4 h of growth cells were harvested and purified using a His-Trap HP Ni^2+^ affinity column by GE Healthcare. The His_8_-tag was removed from the purified protein by digestion at room temperature overnight with TEV protease engineered to contain a non-cleavable His_8_-tag. The resulting cleaved His_8_-tag as well as the TEV protease were removed from Ccs1 by another Ni^2+^ affinity purification.

### Protein metallation

Sod1 was stripped of metals by incubation in Sod1 stripping buffer 1 (50 mM NaOAc pH 3.5, 10 mM EDTA, 10 mM DTT) for 4 h at room temperature and then transferred to metal-free buffer 2 (50 mM NaOAc pH 5.5, 10 mM DTT) overnight at 4 °C. The metal free protein was buffer exchanged into 50 mM Tris pH 8. Ccs1 was stripped of metals by incubation in Ccs1 stripping buffer (20 mM Tris pH 8, 10 mM EDTA, 10 mM DTT) overnight at 4 °C. The metal-free protein was buffer exchanged into either metal free 50 mM Tris pH. All proteins were Cu(I) loaded anaerobically using Cu–PF_6_. Metal loading was confirmed by induced coupled plasma mass spectrometry (ICP-MS) using the Agilent 7900 facility here at UTD. Samples for ICP-MS were digested with 1% HNO_3_ for analysis and performed in triplicate. The buffer in these experiments was measured for baseline metal content. Sod1 activity assays were performed as described previously (Fetherolf et al. 2017a, b).

### Cu(I)-loaded Ctr1c production

Apo-Ctr1c was quantified by amino acid analysis by AAA Service Laboratory, Inc. Absorbance was measured from 200 to 360 nm using a Cary300 UV–Vis spectrophotometer by Agilent and the extinction coefficient was calculated. The Ctr1c-peptide was Cu(I)-loaded anaerobically in equimolar ratio with Cu(I)-(CH_3_CN_4_)PF_6_ for 2 h and excess unbound Cu(I) was removed by size-exclusion chromatography using a BioRad P2 column in spectroscopy buffer or by dialysis. ICP-MS results indicated that one Cu(I) ion was coordinated by 3 Ctr1c peptides, similar to what is seen in full-length Ctr1 (De Feo et al. 2009). Even when copper was added in 3–5 fold excess of peptide, the resulting copper bound remained consistent.

### Amine-specific labeling of Ctr1c

Apo and Cu(I)-Ctr1c were incubated with equimolar amounts of Alexa-488 succinimidyl ester by Fisher at room temperature in pH 7.4 buffer for 1 h. The labeling reaction was quenched with 1 M Tris pH 8 was then dialyzed overnight at 4 °C to remove excess dye and its metal status was confirmed by assaying its ability to provide Cu(I) to Sod1.

### Nickel pulldown assay

His_8_-tagged WT and mutant Ccs1 and Sod1 variants were expressed and purified by Ni-affinity chromatography. 5uM bait protein was incubated with 40 µL Ni-NTA agarose bead slurry in 20 mM Tris pH 7.5, 150 mM NaCl, 2 mM TCEP-HCl, and 200 µM BCS for 1 h at room temperature. 15uM Cu(I)-Ctr1c_488_ was incubated with the bait proteins at room temperature for 1 h and washed three times with 500uL 20 mM Tris pH 7.5, 150 mM NaCl, 2 mM TCEP-HCl, and 100 mM imidazole. Proteins for SDS-PAGE analysis were eluted by the addition of 2X Laemmli dye with BME and boiling. Reactions were visualized on a BioRad 4–20% pre-cast SDS-PAGE stain-free gel using the BioRad ChemiDoc stain-free protocol or fluorescently using the Typhoon 9500 by Thermo. For 384-well plate analysis, proteins were treated as above, then eluted with 40 µL 20 mM Tris pH 7.5, 150 mM NaCl, 2 mM TCEP-HCl, and 1 M Imidazole. 15 µL of each fraction were loaded onto a 384-well clear-bottom microtiter plate and visualized on the Typhoon 9500. Samples from these fractions were also analyzed for metal content by ICP-MS.

### Sod1, Ccs1, and Cu(I)-Ctr1c-Ccs1 affinity determination

H46R/H48Q/C146S Sod1 was fluorescently labeled with Alexa-546-C-maleimide and the reaction was quenched with DTT. Excess unbound dye was removed by gel filtration using a GE Sepharose S200 increase gel filtration column. Final Sod1-probe concentration in all assays was 10 nM. Ccs1 was titrated into reactions in increasing concentrations. We followed the HI-FI method to determine dissociation constants (K_D_) as outlined in previous publications (Winkler et al. 2012). Fluorescence quenching was visualized and K_D_ and binding curves were determined with the GraphPad Prism software suite.

## Results

### Ctr1c stably binds Ccs through a Cu(I) intermediate

**Fig. 1:**
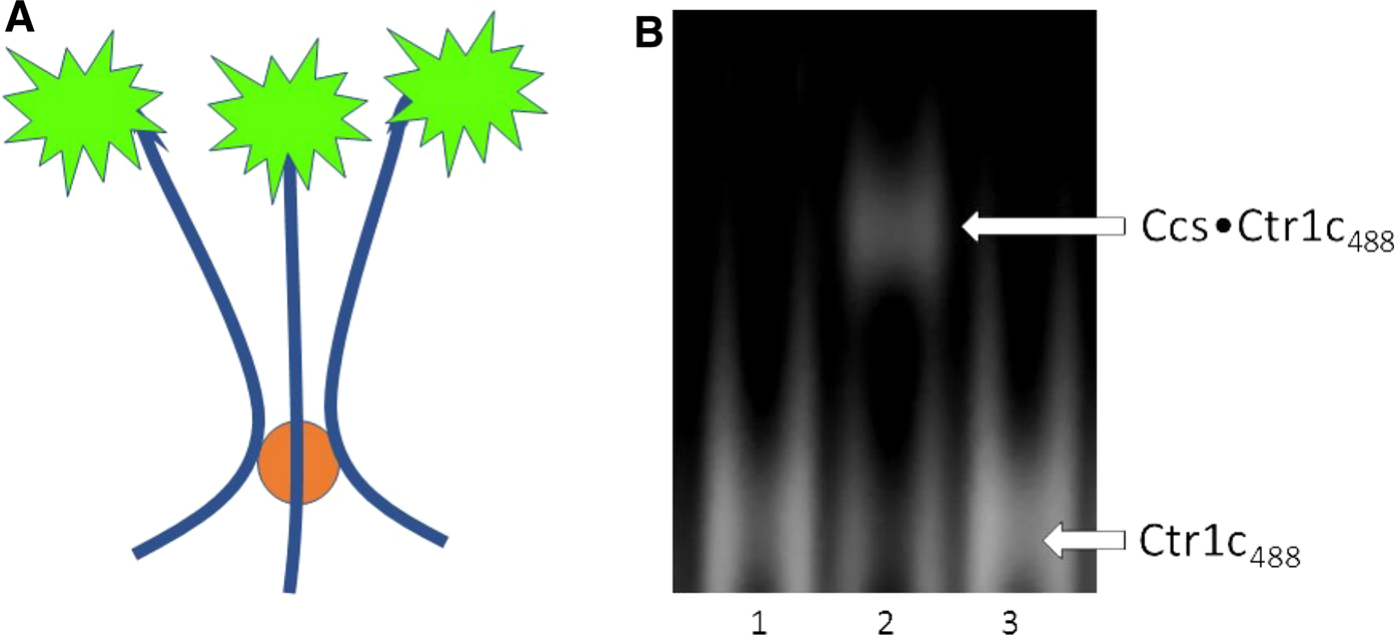
Ctr1c binding to Ccs is copper dependent. **a** Copper (orange circle) is coordinated by three Ctr1c_488_ peptides at their C-terminal HCH motifs. The Alexa 488 dye is conjugated to the N-terminus using a succinimidyl ester linkage. ICP-MS determined that Ctr1c_488_ bound Cu(I) in a 3:1 stoichiometry, similar to full-length Ctr1. **b** Native PAGE gel imaged fluorescently for Alexa 488. Lane 1: Ctr1c_488_ + Ccs. Lane 2: Cu(I) − Ctr1c_488_ + Ccs. Lane 3: Cu(I) − Ctr1c_488_ + E,E-Sod1^SH^. (Color figure online)

To focus upon the intracellular copper transfer between Ctr1 and Ccs, we utilized the cytosolic copper delivery domain of Ctr1 (Ctr1c) (Kahra et al. 2016). Ctr1c is a short 13 amino acid peptide that is difficult to visualize/quantitate biochemically, so a fluorescent label (Alexa 488) was conjugated to the N-terminus. Labeled peptide (Ctr1c_488_) was purified and then visualized on Native-PAGE to confirm that non-native oligomerization did not occur (Fig. S1). Fluoro-labeling did not affect copper binding, as both labeled and unlabeled Ctr1c peptides bound Cu(I) equivalently as determined by ICP-MS analysis (Table 1). The percentage of Cu(I) bound to Ctr1c indicates that multiple Ctr1c peptides bind a single copper at their HCH motifs, likely in a 3:1–4:1 stoichiometry (Fig. 1a). We suggest that the 3:1 ratio is more likely as Cu(I) prefers to bind 2 or 3 ligands. Trimeric binding of Ctr1c to copper also occurs in full-length Ctr1. To determine if Ctr1c and Ccs would stably interact, both apo and Cu(I)-loaded Ctr1c_488_ were incubated with Ccs and visualized fluorescently on Native-PAGE. Apo-Ctr1c_488_ did not bind to Ccs, but Cu(I)-Ctr1c_488_ formed a stable complex that ran as a clear band on the gel (Fig. 1b, Lanes 1 and 2). However, Ctr1c could not form a similar interaction with Ccs’ target (metal-free, disulfide reduced Sod1 (E,E-Sod1^SH^)). Incubation of Cu(I)-Ctr1c_488_ with E,E-Sod1^SH^ did not result in stable complex formation (Fig. 1b, Lane 3). Furthermore, the Sod1 from this reaction was subsequently analyzed for metal content by ICP-MS, and no direct copper transfer occurred between Ctr1c and Sod1.

**Table 1:** ICP-MS results shown as the percentage of copper-bound protein

ICP-MS results		Protein:Cu(I) ratio
Ctr1c	22.6% ± 1.4	3–4:1
Ctr1c_488_	23.9% ± 1.7	3–4:1
WT Ccs	87.4% ± 2.6	1:1
MXAXXA	85.2% ± 0.4	1:1
AXA	82.0% ± 1.2	1:1

The first two rows show the copper loading efficiency of the Alexa 488 labeled and non-labeled Ctr1c constructs. Percent bound remained consistent regardless of the amount of excess copper added. Final three rows describe the amount of copper bound to Ccs constructs after Ni pulldown assays with Cu(I)-loaded Ctr1c_488_

### Stable Ctr1c·Ccs interaction can form at either copper binding motif of Ccs

**Fig. 2:**
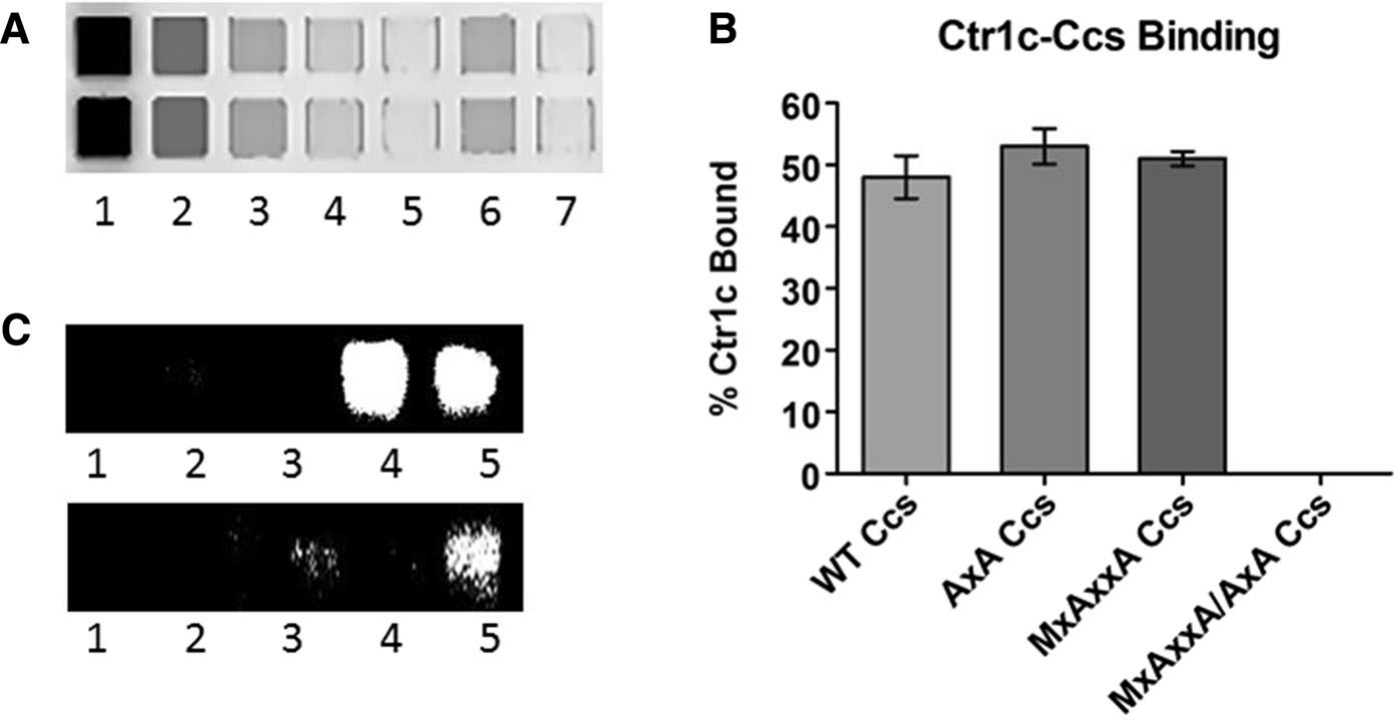
Ctr1c can bind both copper binding domains of Ccs. **a** Nickel pulldown assays were loaded into a clear-bottom 384-well plate and visualized fluorescently using the Typhoon 9500. Lane 1: Input, Lane 2: Flow-through, Lanes 3-5: Washes, Lane 6: Elution, Lane 7: Buffer blank. **b** This graph of fluorescence intensity is normalized to mutant Ccs lacking copper binding motifs. (**c**, Top) Lane 1: Sod1 stripped of metals, Lane 2: Sod1 with Cu(I) − Ctr1c, Lane 3: Sod1 with apo-MxAxxA Ccs, Lane 4: Sod1 with apo-MxAxxA Ccs and Cu(I)-Ctr1c, Lane 5: Sod1 with Cu(1)- WT Ccs. (C, Bottom) Lane 1: Sod1 stripped of metals, Lane 2: Sod1 with Cu(I)-Ctr1c, Lane 3: Sod1 with apo-AxA Ccs, Lane 4: Sod1 with apo-AxA Ccs and Cu(I)-Ctr1c, Lane 5: Sod1 with Cu(1)-WT Ccs

We use a high-throughput experimental setup that takes advantage of clear-bottom microplates for fluorescent-based visualization and quantification of pull-down results (detailed in the “Materials and Methods” section). Figure 2a shows an example of the pull-down assays performed to examine the role of the copper binding motifs in Ccs in coordinating Ctr1c. His-tagged wild-type Ccs or Ccs variants with Cu(I)-Ctr1c_488_ (top row) were assayed and then normalized against a copper-null variant of Ccs that was also incubated with Cu(I)-Ctr1c_488_ (bottom row). Three experimental replicates were performed and quantified to determine relative binding propensity (Fig. 2b). There does not appear to be a strong preference for either copper binding site in Ccs (D1–MxCxxC or D3–CxC). The copper-mediated interaction between Ctr1c and Ccs occurs as long as copper is bound by at least one of these sites. Similar Cu(I) content was observed in all three samples by ICP-MS (Table 1). Incubating with 15 µM Cu(I)-Ctr1c_488_ consistently resulted in the recovery of > 4 µM copper-bound Ccs, supporting the binding of copper by peptide in a 3:1 ratio. If the peptide to copper ratio was 4:1 the maximum amount of copper transferred to Ccs would be 3.75 µM. In vitro Sod1 activation assays performed using the Cu(I)-Ctr1c_488_·Ccs containing samples showed that the complexes are functional and although Cu(I)-Ctr1c_488_ will coordinate with both copper-binding motifs in Ccs, only the CxC motif in Ccs D3 is critical for full activation of immature Sod1 under these conditions (Fig. 2c).

### Copper-loaded Ctr1c associates stably with a Ccs·Sod1 complex

**Fig. 3:**
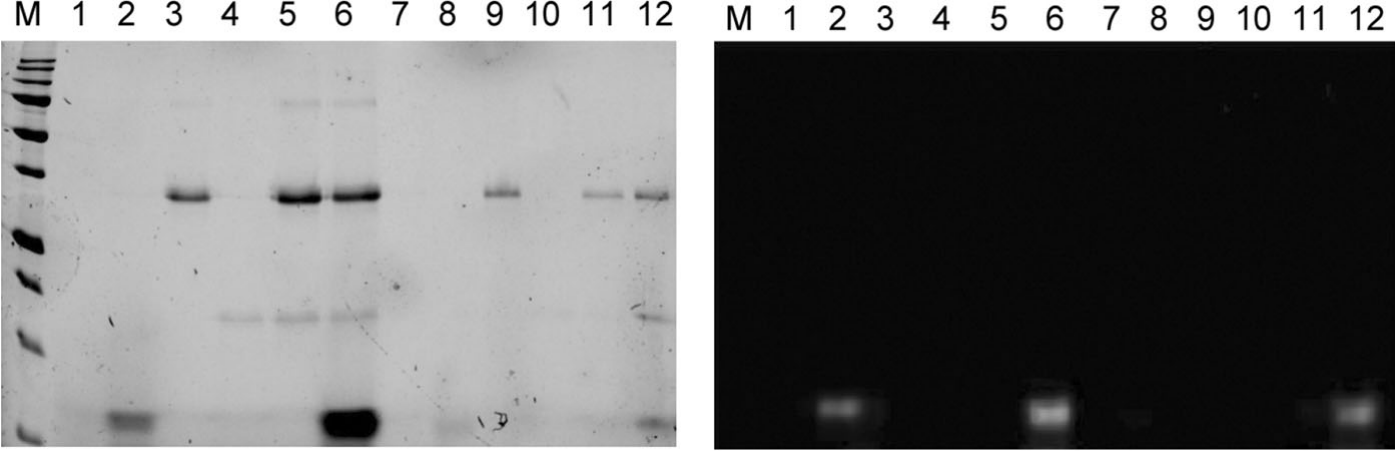
Immature Sod1 can form a stable Sod1·Ccs·Ctr1c heterotrimeric complex. Lane 1: beads supernatant, Lane 2: Cu(I)-Ctr1c_488_ supernatant, Lane 3: His_8_-Ccs supernatant, Lane 4: Sod1 supernatant, Lane 5: His_8_-Ccs·Sod1 supernatant, Lane 6: His_8_-Ccs·Sod1 and Cu(I)-Ctr1c_488_ supernatant, Lane 7: beads elution, Lane 8: Cu(I)-Ctr1c_488_ elution, Lane 9: His_8_-Ccs elution, Lane 10: Sod1 elution, Lane 11: His_8_-Ccs·Sod1 elution, Lane 12: His_8_-Ccs·Sod1 and Cu(I)-Ctr1c_488_ elution. The right image is the same gel as the left (stain free), but visualized fluorescently

**Fig. 4:**
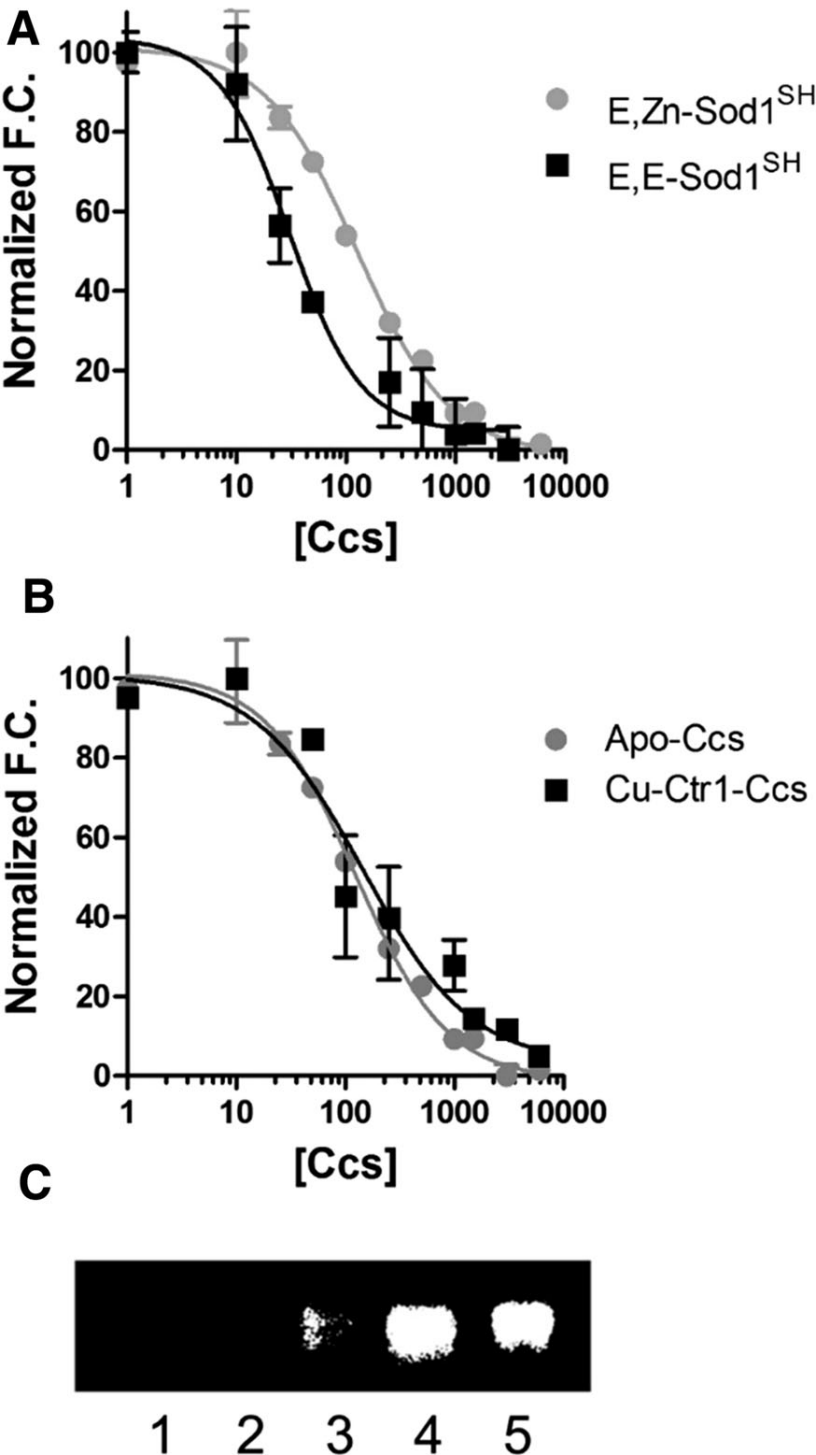
Cu(I)-Ctr1c binding to Ccs does not affect its Sod1 binding affinity. The concentration of Ccs1 is nanomolar. The curves have been normalized for comparison for panels **a** and **b**. **a** apo-Ccs binding to completely immature Sod1 (89 nM ± 11) and Zn-bound Sod1 (22 nM ± 6). **b** Comparison of binding affinities for apo-Ccs (89 nM ± 11) and Cu(I)-Ctr1c complexed Ccs to Sod1 (120 nM ± 27). **c** Sod1 activity assay. Lane 1: Sod1 stripped of metals, Lane2: Sod1 with Cu(I)-Ctr1c, Lane3: Sod1 with apo-Ccs, Lane4: Sod1 with Cu(I)-Ctr1c complexed Ccs, Lane 5: Sod1 with Cu(I)-Wt Ccs

To characterize possible Ctr1c·Ccs interactions with Sod1, similar pull-down assays utilizing a His-tagged Ccs in complex with Cu(I)-Ctr1c_488_ were conducted (Fig. 3). Immediate copper transfer and dissociation does not occur and a stable Cu(I)-Ctr1c_488_·Ccs complex is formed, as previously demonstrated (Fig. 1). The Sod1 molecule used in this assay is an engineered immature form of Sod1 that cannot bind copper at the active site or form its intra-subunit disulfide bond (X,Zn-Sod1^X^), which is commonly used to form “stalled” Ccs·Sod1 complexes (Lamb et al. 2000, 2001; Winkler et al. 2009). Direct interaction between Cu(I)-Ctr1c_488_ and X,Zn-Sod1^X^ is not detected under any conditions tested (Figs. 1b, 2c). In addition, we show that a stable Cu(I)-Ctr1c_488_·Ccs complex can recognize and bind to X,Zn-Sod1^X^ in a similar manner to that of Ccs alone (Fig. 4b). Strikingly, Cu(I)-Ctr1c_488_ also interacts stably with a pre-formed Ccs·X,Zn-Sod1^X^ heterodimeric complex (Fig. 3, lane 12). The Ctr1c molecule does not dissociate upon Ccs binding to Sod1 and forms a Cu(I)-Ctr1c_488_·Ccs·X,Zn-Sod1^X^ heterotrimeric complex, supporting multiple delivery options arising from the Ctr1 transporter.

### Ccs association with Ctr1c does NOT affect its affinity for Sod1

The assumed order of operation for Sod1 activation starts with the metalation of Ccs, which then finds/binds an immature Sod1 molecule to activate (see reviews Culotta, Lin et al. 1999; Rosenzweig 2001; Furukawa and O’Halloran 2006; Leitch et al. 2009; Robinson and Winge 2010; Fetherolf et al. 2017a, b). Much like the yeast Ccs·Sod1 interaction (Boyd et al. 2018), human Ccs favors binding to completely immature Sod1 (E,E-Sod1^SH^) (Fig. 4a). The copper occupancy of Ccs has a negligible role in Sod1 binding affinity, as the copper-loaded form of Ccs binds to E,E-Sod1^SH^ in a nearly indistinguishable fashion. Additionally, a pre-made Cu(I)-Ctr1c·Ccs complex binds E,E-Sod1^SH^ with a similar affinity to both apo and copper-bound Ccs (Fig. 4b). Again, we also wanted to ensure that the Cu(I)-Ctr1c·Ccs complex assembled is functional and can fully activate immature Sod1. In vitro Sod1 activation assays reveal that Cu(I)-Ctr1c cannot directly activate Sod1, alone, but requires the Cu(I)-Ctr1c·Ccs complex (Fig. 4c, Lanes 4 and 2, respectively). Control gels of the reactions were visualized by multiple approaches to confirm equal loading (Fig. S2). Our results continue to support the idea that an entire copper-trafficking pathway can, but is not necessitated to occur within a single complex, yet the question arises as to what finally triggers eventual dissociation.

### Complete Sod1 activation terminates the Sod1·Ccs·Ctr1c association

**Fig. 5:**
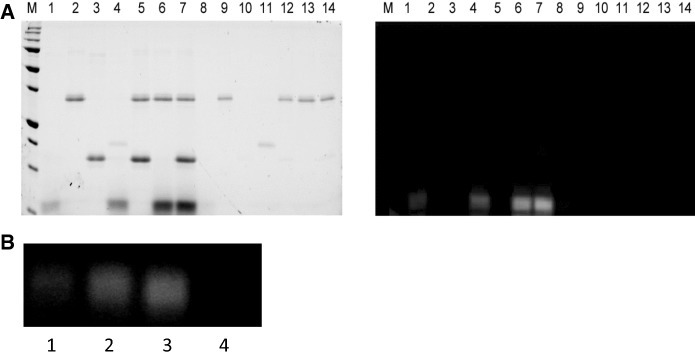
Sod1 maturation disassociates the Sod1·Ccs·Cu(I)-Ctr1c complex. Lane 1: Cu(I)-Ctr1c_488_ supernatant, Lane 2: His_8_-Ccs supernatant, Lane 3: Sod1 supernatant, Lane 4: His_8_-Sod1 with Cu(I)-Ctr1c_488_ supernatant, Lane 5: His_8_-Ccs·Sod1 supernatant, Lane 6: His_8_-Ccs with Cu(I)-Ctr1c_488_ supernatant, Lane 7: His_8_-Ccs·Sod1 with Cu(I)-Ctr1c_488_ supernatant, Lane 8: Cu(I)-Ctr1c_488_ elution, Lane 9: His_8_-Ccs elution, Lane 10: Sod1 elution, Lane 11: His_8_-Sod1 with Cu(I)-Ctr1c_488_ elution, Lane 12: His_8_-Ccs·Sod1 elution, Lane 13: His_8_-Ccs with Cu(I)-Ctr1c_488_ elution, Lane 14: His_8_-Ccs·Sod1 with Cu(I)-Ctr1c_488_ elution. The right image is the same gel as the left (stain free), but visualized fluorescently. **b** Lane 1: Cu(I)-loaded Ctr1c_488_ + WT Sod1 from **a**, Lane 4. Lane 2: Cu(I)-loaded WT His_8_-Ccs + WT Sod1 set up separately as a positive control. Lane 3: WT His_8_-Ccs + WT Sod1 + Cu(I)-Ctr1c_488_ from **a**, Lane 7. Lane 4: WT His_8_-Ccs + immature Sod1 + Cu(I)-Ctr1c_488_ from Fig. 3, Lane 12

To study dissociation of the Cu(I)-Ctr1c·Ccs·Sod1 complex, we performed pulldowns now using immature Sod1 (E,Zn-Sod1^SH^) (Fig. 5a). When E,Zn-Sod1^SH^ was added to a pre-formed Cu(I)-Ctr1c_488_·Ccs complex, immediate and complete dissociation of the complex resulted (Fig. 5a, Lane 14). Activation assays were performed on samples obtained from the pulldowns and the E,Zn-Sod1^SH^ that had been allowed to interact with Cu(I)-Ctr1c·Ccs complex was analyzed and had activity comparable to that of E,Zn-Sod1^SH^ incubated with Cu(I)-Ccs, alone (i.e. the Sod1 is now fully mature (Cu,Zn-Sod1^SS^)). E,Zn-Sod1^SH^ incubated with Cu(I)-Ctr1c alone did not have activity, nor did the immaturely-trapped X,Zn-Sod1^X^ mutant that was complexed with Cu(I)-Ctr1c·Ccs (Fig. 5b). This clearly indicates that the Cu(I)-Ccs·Ctr1c complex can activate immature Sod1 in a way that Cu(I)-Ctr1c cannot accomplish (Fig. 4c, Lanes 4 and 2, respectively). Only complete Sod1 activation triggers dissociation of the stable Cu(I)-Ctr1c·Ccs·Sod1 complex, suggesting that the portion of Sod1 activated through this direct Ctr1-to-Ccs-to-Sod1 pathway occurs while tethered to the cell membrane via the complete Ctr1 Cu-transporter.

## Discussion

Up until now, a considerable amount of work has been completed on the Ccs-mediated Sod1 activation process (reviewed in Seetharaman et al. 2009), yet details of copper transfer to Ccs were still unclear. Our data shows that the intracellular C-terminal tail of the influx transporter Ctr1 (Ctr1c) forms a stable copper-mediated interaction with Ccs, as complete copper transfer does not immediately follow first contact. The introduction of an engineered Sod1 variant that cannot bind copper or form its conserved intra-subunit disulfide bond (X, Zn-Sod1^X^) generates a heterotrimeric Cu(I)-Ctr1c·Ccs·Sod1 complex. Only complete Sod1 activation severs the ties between transporter, chaperone, and target. Together, the results presented above provide clear evidence for a complete copper delivery pathway held within a singular 3-part complex.

The Unger laboratory was the first to show that yeast Ccs1 interacts directly with Ctr1 (Pope et al. 2013). Their work highlighted that yCcs1 can engage with lipid bilayers and suggested that recruitment to the plasma membrane is an essential step in Ccs-mediated Sod1 activation (Pope et al. 2013). The authors of that study submit mechanistic ambiguities in the proposed process and how further investigation would be needed to “unravel” these details. In the present study, we have taken a much narrower focus upon the intracellular C-terminal domain of Ctr1 (Ctr1c). This domain is known to be important for the coordination and eventual distribution of copper to trafficking molecules within the cytosol (Xiao and Wedd 2002; Kahra et al. 2016). We show that this domain alone is enough to interact with Ccs or a Ccs·Sod1 complex, and the interactions are entirely copper dependent (Figs. 1, 4), much like for the complete Ctr1 transporter (Pope et al. 2013). Similar results have been demonstrated for Ctr1c and the copper chaperone Atx1 (Xiao and Wedd 2002), which shares similarity with D1 of Ccs.

Numerous groups, including our own, have previously shown that Ccs recognizes and binds immature forms of Sod1 (Banci et al. 2012; Wright et al. 2016; Fetherolf et al. 2017a, b; Luchinat et al. 2017; Boyd et al. 2018). The Sod1-like D2 of Ccs directs this interaction with D3 contributing to high-affinity binding and full activation (Boyd et al. 2018). More recently, we have demonstrated that copper binding by Ccs does not significantly affect the Sod1 interaction (Boyd et al. 2018). The question then arose as to whether the Cu(I)-Ctr1c·Ccs interaction alters Ccs recognition and binding to Sod1. Upon examination, Ccs can recognize and bind immature Sod1 while still coordinating copper with Ctr1c. In fact, a stable high-affinity Cu(I)-Ctr1c·Ccs·Sod1 complex is observed (Figs. 2, 3, 4). The next logical steps were to test if this heterotrimeric complex results in activation of Sod1.

Ccs-mediated Sod1 activation is an intricate process involving fold-induced zinc binding by Sod1 that is followed by disulfide bond driven copper delivery (Culotta et al. 1997, 2006). Multiple lines of evidence suggest that cellular conditions may necessitate the role(s) for Ccs and that ancillary molecules like reduced glutathione (GSH) may facilitate the activation process (Maryon et al. 2013). In fact, GSH has been shown to promote an entirely Ccs-independent Sod1 activation pathway in mammals (Leitch et al. 2009). The Cu(I)-Ctr1c construct will not donate Cu(I) to Sod1 directly, but a preformed Cu(I)-Ctr1c·Ccs complex can fully activate immature Sod1 in vitro (Fig. 5b). This excludes a Ccs-independent mechanism of Sod1 activation catalyzed directly by Ctr1c.

**Fig. 6:**
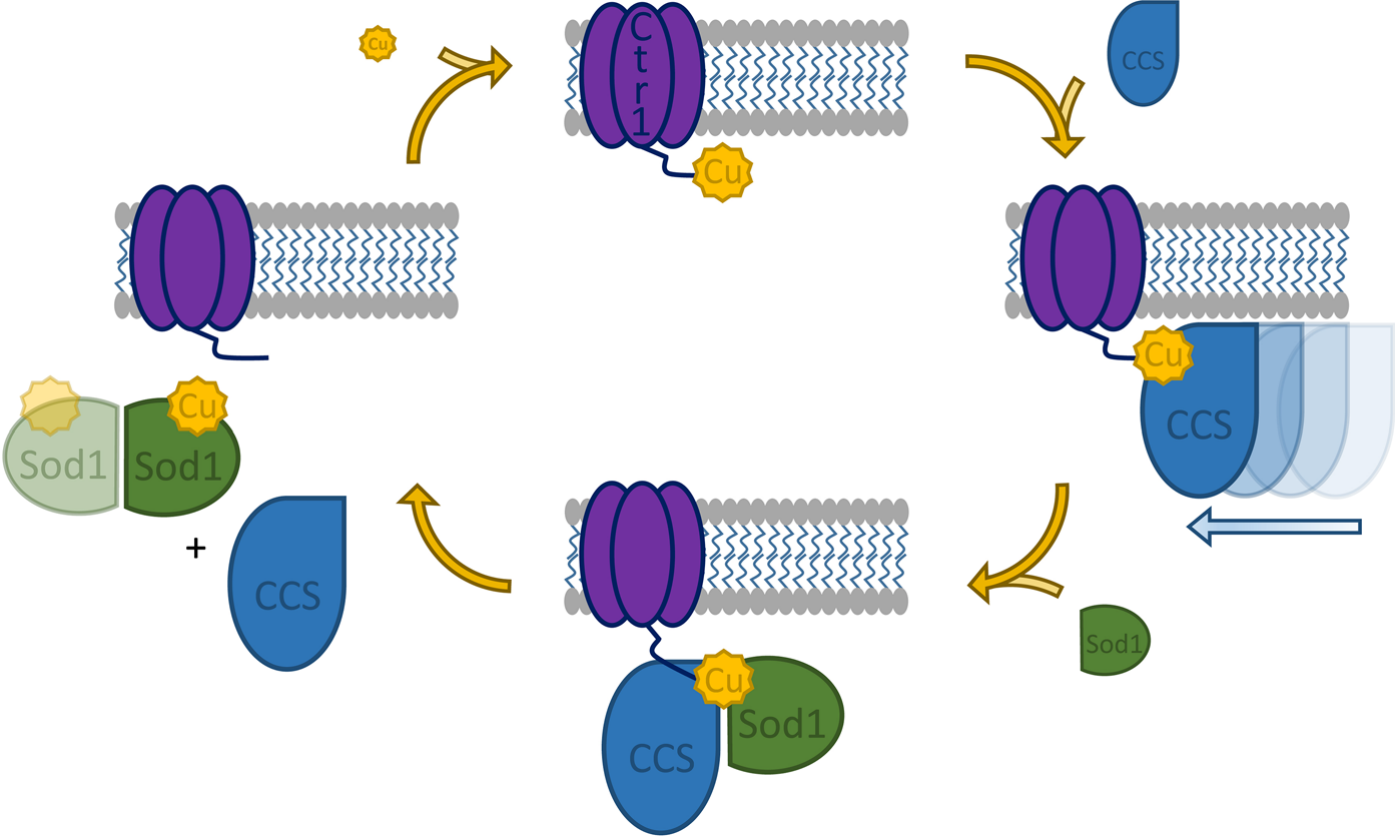
Model of Ccs copper acquisition from Ctr1 and complex disassembly by Sod1 maturation. Ctr1 (purple) transports copper (yellow) into the cytosol. Ccs (blue) scans the membrane and associates with the copper-bound Ctr1 C-terminal domain. Sod1 (green) interacts with the Cu(I)-Ctr1-Ccs complex. Sod1 maturation terminates the ternary complex. Ccs is allowed to cycle through the copper-acquisition process again. The Sod1 homo-dimer is fully active. (Color figure online)

It is quite intriguing that the Cu(I)-Ctr1c·Ccs·Sod1 complex is not transiently associated. This suggests that a simple Cu(I) hand-off between Ctr1c and Ccs is not occurring; so, what induces eventual dissociation? The Ccs·Sod1 interaction, without Ctr1c, has also been shown to be remarkably stable as long as copper cannot be delivered to the Sod1 active site (Fetherolf et al. 2017a, b). This can be ensured in two ways: (1) copper is not provided to the complex or (2) the active site is modified so that the bound copper cannot be delivered to that site (Fetherolf et al. 2017a, b). We have previously shown that Ccs delivers copper to an “entry-site” near the Sod1·Ccs interface (Fetherolf et al. 2017a, b). Oxygen dependent disulfide bond formation eliminates this site, promotes copper shuttling to the nearby active site, and terminates interaction with Ccs (Fetherolf et al. 2017a, b). Copper must be present to form the Cu(I)-Ctr1c·Ccs·Sod1 heterotrimer, but the Sod1 active site must be ablated or the complex readily activates Sod1 and the complex promptly dissolves. This likely indicates that Ctr1c is co-coordinating the copper ion up until disulfide bond formation in Sod1 disperses the complex producing Cu,Zn-Sod1^SS^ that can now homodimerize along with copper-free forms of both Ccs and Ctr1c that no longer have any affinity for each other (Fig. 6).

Noteworthy is that Pope et al. showed that yeast Ccs1 strongly associates with lipid bilayers, but that the yCcs1·ySod1 heterodimer reduced this propensity most likely due to a critical positively charged patch on yCcs1 that is not present on ySod1 (Pope et al. 2013). This observation may explain how Ccs first comes into contact with Cu(I)-Ctr1, and how the products of this reaction (e.g. activated Sod1 and apo-Ccs) are provided release from the membrane to freely access other cellular locations. Together, this present study establishes an all-inclusive design for Ctr1-to-Ccs-to-Sod1 copper delivery and provides new evidence for a singular heterotrimeric complex forming the foundation for the pathway.

## Electronic supplementary material

Below is the link to the electronic supplementary material.

Supplementary material 1 (DOCX 93 kb)
