# Supplementary material for: Copper–zinc superoxide dismutase (Sod1) activation terminates interaction between its copper chaperone (Ccs) and the cytosolic metal-binding domain of the copper importer Ctr1
Source: Biometals. 2019 Jul 10;32(4):695–705. doi: 10.1007/s10534-019-00206-3 (PMC6647829; doi:10.1007/s10534-019-00206-3)
Supplement: Supplementary file 1 — Supplementary material 1 (DOCX 93 kb) [file 10534_2019_206_MOESM1_ESM.docx]

**Supplementary Figure 1.** N-terminal fluorescent labeling of Ctr1c with Alexa 488 succinimidyl ester. Ctr1c was labeled to allow for visualization. In this fluor-image unlabeled Ctr1c cannot be seen (left lane), but increasing titrations of Alexa 488-conjugated peptide are visualized.

**Supplementary Figure 2.** Activity gel loading control. N-terminal fluorescent labeling of Ctr1c with Alexa 488 succinimidyl ester allows for visualization. Lane 1: apo-Sod1 alone, Lane 2: apo-Sod1 and Cu(I)-Ctr1c, Lane 3: apo-Sod1 and apo-Ccs, Lane 4: apo-Sod1, apo-Ccs, and Cu(I)-Ctr1c, Lane 5: apo-Sod1 and Cu(I) Ccs. (A) is a Coomassie image of the SDS-PAGE gel, and (B) is the same gel imaged using a stain-free method. Fluorescently labeled peptide only appears in the stain-free image and runs with the dye-front. Coomassie blue dye interferes with fluorescent signaling.


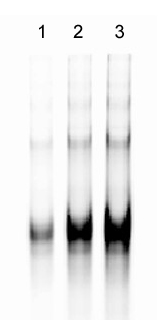


**Supplementary Figure 1.**


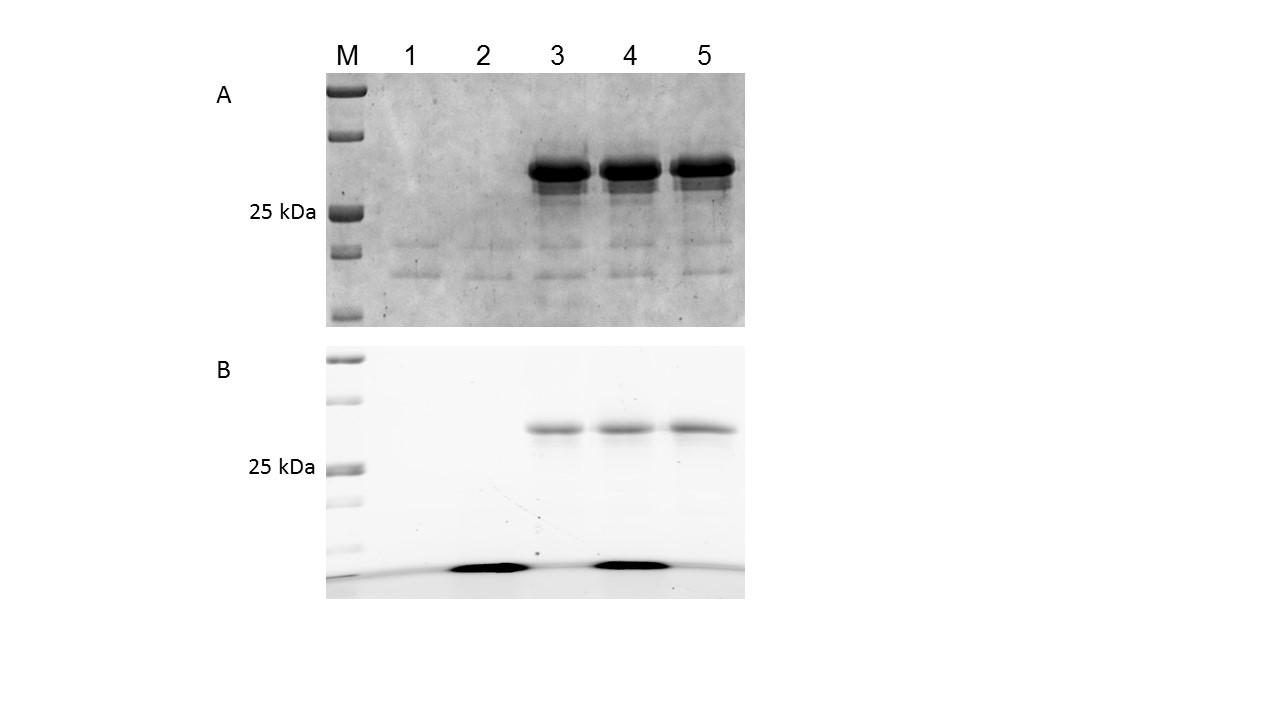


**Supplementary Figure 2.**
